# Molecular dynamics of chemotactic signalling orchestrates dental pulp stem cell fibrosis during aging

**DOI:** 10.3389/fcell.2024.1530644

**Published:** 2025-01-10

**Authors:** Tianmeng Sun, Qing Zhong, Xiaoyi Yu, Huanyu Luo, Feilong Ren, Cangwei Liu, Peng Chen, Fabian Flores-Borja, Hongchen Sun, Zhengwen An

**Affiliations:** ^1^ Department of Oral Biology, School and Hospital of Stomatology, Jilin University, Changchun, China; ^2^ Key Laboratory of Tooth Development and Bone Remodeling of Jilin Province, School and Hospital of Stomatology, Jilin University, Changchun, China; ^3^ Department of Oral Pathology, School and Hospital of Stomatology, Jilin University, Changchun, China; ^4^ Department of Genetics, College of Basic Medical Sciences, Jilin University, Changchun, China; ^5^ Centre for Oral Immunobiology and Regenerative Medicine, Barts and the London School of Medicine and Dentistry, Blizard Institute, Queen Mary University of London, London, United Kingdom

**Keywords:** dental pulp aging, fibrosis, single cell analysis, chemotactic signals, immune-fibroblast axis

## Abstract

Aging often triggers dental pulp fibrosis, resulting in clinical repercussions such as increased susceptibility to dental infections, compromised tooth vitality, and reduced responsiveness to dental interventions. Despite its prevalence, the precise molecular mechanisms underlying this condition remains unclear. Leveraging single-cell transcriptome analysis from both our own and publicly available datasets, we identified Ccrl2^+^ macrophages as particularly vulnerable during the early stages of aging. Notably, dental pulp progenitors with high expression of RARRES2, a unique ligand for CCRL2, facilitate the selective recruitment of a specific macrophage population to the stem cell niches. This process culminates in the formation of the ligand-receptor complex that engages CMKLR1, a receptor broadly expressed across macrophage populations. This interaction drives macrophage activation and expansion through the RARRES2/CCRL2/CMKLR1 axis. Through rigorous experimental validation, we demonstrated that macrophage activation and expansion within stem cell niches lead to increased secretion of proinflammatory factors, promoting dental pulp fibrosis during aging. Our findings uncover the intricate molecular dynamics of dental pulp aging, emphasizing immune microenvironment interactions. This study provides a novel perspective on potential therapeutic strategies for age-related pulp diseases by targeting macrophages and modulating the immune microenvironment.

## 1 Introduction

Aging is an inevitable and gradual biological process that progressively affects the structural and functional integrity of the body over time (Partridge et al., 2018). These changes increase the risk of age-related conditions such as Alzheimer’s disease, diabetes, and endocrine disorders (Borghesan et al., 2020; Bao et al., 2023). Dental pulp stem cells, like stem cells in other tissues, undergo degenerative changes with age, leading to impaired tissue function, reduced self-regulation of the internal environment, and heightened vulnerability to environmental stressors (Bao et al., 2023). However, the intricate biological mechanisms underlying the aging process, particularly in dental pulp tissue, remail poorly understood.

Immune cells, as the initial sentinels of defence, play a pivotal role alongside stem cells in tissue regeneration and repair. Recent studies highlight on the critical role of immune cells in aging, influencing both health maintenance and the progression of age-related diseases. Macrophages, in particular, exhibit age-dependent alterations in gene expression, which modulate their functionality in response to environmental cues (Oishi and Manabe, 2016). Depending on these cues, macrophages can differentiate into pro-inflammatory (M1-type) and anti-inflammatory (M2-type) phenotypes, which are indispensable for immune responses (Bao et al., 2023) and tissue repair processes (Hirayama et al., 2017). Macrophages express chemokine (C-C motif) receptor-like 2 (CCRL2), a receptor with a seven-transmembrane structural domain that regulates inflammatory responses in various clinical contexts and is significantly upregulated by inflammatory signaling (Al Delbany et al., 2021). CCRL2 deficiency reduces pro-inflammatory cytokine production and NF-κB signaling (Yin et al., 2021). Elevated levels of inflammation, driven by increased pro-inflammatory factors, have been implicated in the onset of fibrosis across multiple tissues and diseases. For instance, macrophage-derived molecules such as CXCL2, CCL2, and IL-1β have been linked to the progression of Idiopathic Pulmonary Fibrosis (Nie et al., 2023; Trachalaki et al., 2021). In myocardial fibrosis mouse models, medications targeting IL-1β and IL-18 expression effectively reduced fibrosis (Gao et al., 2021). Similarly, skin and lung fibrosis worsened in wild-type mice following the transplantation of leukocytes with high expression of IL-1β and TNF-α (Sawada et al., 2021). In mouse liver models, inhibiting macrophage secretion of CXCL2 or utilizing CXCR2 inhibitors mitigated fibrosis (Ge et al., 2021).

The dental pulp consists of diverse cell types with distinct roles and regulatory mechanisms intricately associated with tooth growth, rejuvenation, and immune responses (Ren et al., 2022). Age-related changes in pulp tissue include a reduction in fibroblast numbers, altered dentin-forming cell morphology, decreased neurovascular density, metabolic dysregulation, and impaired defensive and reparative functions. These changes collectively culminate in pulp fibrosis (Maeda, 2020) characterized by excessive fibrous connective tissue within the pulp, which significantly compromise oral health and function. Despite its frequent clinical observation, the molecular mechanisms underlying pulp fibrosis remain incompletely elucidated.

Conventional RNA sequencing provides aggregate characteristics of tissues and cell populations but lacks the relations to identify cell-specific distinctions and heterogeneity. In this study, we utilize the advantages of single-cell technology (Hedlund and Deng, 2018) to unravel the intrinsic molecular mechanisms and intercellular communication within dental pulp during early stages of aging. It offers novel insights into stem cell aging and suggests potential strategies to address age-related diseases.

## 2 Materials and methods

### 2.1 Sample collections for single cell RNA-sequencing

Mandibles were carefully dissected from 15 C57BL/6 mice aged 8–12 months, representing the early stages of aging, using precise dissection techniques under a stereomicroscope. Surrounding bones and soft tissues were meticulously removed with scalpels and fine-point tweezers. The incisor pulp, with minimal inclusion of dental epithelium, was isolated, fragmented, and immersed in cold PBS. The pulp fragments were enzymatically digested with collagenase in α-MEM (Gibco) at 37°C for 30–45 min. The resulting single cell suspension was quantified and loaded into the 10X Chromium system, targeting approximately 10,000 cells for barcoding. ScRNA-seq libraries were prepared using the Single Cell 5′Library Kit (10X Genomics). A total of 13,187 cells were successfully barcoded and their transcriptomes were sequenced using the Illumina Novaseq System (Illumina, San Diego, CA).

### 2.2 Single cell RNA-seq data analysis

Raw fastq files from scRNA-seq were mapped to *Mus musculus* assembly GRCm38 (mm 10) using CellRanger (7.0.1). Raw read counts were processed and analyzed using the Seurat R package (4.4.0). Low quality cells were filtered using following parameters: nFeature_RNA > 500, percent. mt < 20, nCount_RNA > 500, log_10_GenesPerUMI > 0.8 (Calculated by log_10_ (nFeature_RNA)/log_10_ (nCount_RNA), representing cell complexity). The compared aged samples with younger controls, we integrated the sequencing data of mouse incisor dental pulp (aged 2–4 months) from the publicly available dataset GSE146123 using Harmony package. Cell clusters were annotated based on significantly differential expressed genes calculated using the Findallmarkers function in Seurat. Cluster interaction relationships and their strengths were inferred using the CellChat (version 1.6.1) package with the complete CellChatDB.mouse database. Additionally, a receptor-ligand pair, RARRES2-CCRL2, identified from reference studies, was manually added to database. Cell differentiation trajectory analysis was performed using the Monocle (version 2.26.0) package, which leverages cell gene expression kinetics. Pseudotime values for each cell, computed using the Monocle algorithm, were visualized on a UMAP plot.

### 2.3 Animal work

All animal work was carried out under the guidelines of the Laboratory Animal Ethics Committee, School of Basic Medical Sciences, Jilin University, China, in compliance with the Declaration of Helsinki, as per license number 2023491. C57BL/6 mice were provided unrestricted access to food and water. They were housed in a specific pathogen-free (SPF) environment, maintained at a stable temperature of 22°C and 55% ± 10% humidity, under a consistent 12-h light-dark cycle. In the study, mice aged 6–8 weeks were categorized as the young adult group, while those aged 8–12 months were classified as the aged group.

### 2.4 RNA extraction and RT-qPCR analysis

Total RNA was extracted from incisor dental pulp tissue using the RNAeasy™ Animal RNA Isolation Kit with Spin Column (Beyotime, R0027). RNA purity and integrity were assessed using the 260/280 absorbance ratio measured with a NanoDorp spectrophotometer (Thermo Fisher Scientific). cDNA synthesis was performed using a reverse transcription kit (YEASEN, 11141ES60) with gene-specific primers. Quantitative real-time PCR was conducted using Hieff qPCR SYBR Green Master Mix (YEASEN, 11202ES03) on a Biorad Real-Time Fluorescent Quantitative PCR Detection System. Gene expression levels were normalised to ACTB mRNA as an internal control, and relative gene expression was calculated using the 2^−ΔΔCT^ method. At least three independent experiments were performed for each analysis. The qPCR primers used in this study are as follow:

*Ccrl2*-F:GCCCCGGACGATGAATATGAT; *Ccrl2*-R:CACCAAGATAAACACCGCCAG.
*Cmklr1*-F:GCCAACCTGCATGGGAAAATA; *Cmklr1*-R:GTGAGGTAGCAAGCTGTGATG.
*Rarres2*-F:CCCTGAGAACCAAATAAGCCCT;*Rarres2*-R:AGCCTGGAGTTGAAAGTCTCTG.
*IL-1β*-F:GCAACTGTTCCTGAACTCAACT; *IL-1β*-R:ATCTTTTGGGGTCCGTCAACT.
*Cxcl2*-F:GAAGTCATAGCCACTCTCAAGG; *Cxcl2*-R:CCTCCTTTCCAGGTCAGTTAGC.
*Actb*-F:TGGAATCCTGTGGCATCCATGAAAC; *Actb*-R:TAAAACGCAGCTCAGTAACAGTCCG.

### 2.5 Western blot

Mice aged 8 weeks were classified as adult mice, while those aged approximately 12 months were classified as elderly. Pulp tissues were harvested from both groups, and an appropriate volume of lysis buffer was added to lyse the tissues. After centrifugation, the supernatant was collected, and protein concentration was determined. The protein sample was mixed with the loading buffer and separated by SDS-PAGE electrophoresis based on their molecular weight. After electrophoresis, the protein was transferred to a PVDF membrane using a wet transfer method. The membrane was blocked with blocking solution for 1 h at room temperature to prevent nonspecific binding. The membrane was incubated overnight at 4°C with the following primary antibodies: β-actin (20536-1AP,Proteintech, 1:2,500), IL-1β (26048-1-AP, Proteintech, 1:1,000), RARRES2 (10216-1-AP, Proteintech, 1:300), CXCL2 (16325-1-AP, Proteintech, 1; 1,000), CMKLR1 (Bioss,bs-10185R, 1:1,000), CCRL2 (BD-PT0714, Biodragon, 1:1,000). After incubation, the membrane was washed several times with TBST to remove unbound primary antibody. The corresponding HRP-conjugated secondary antibody (SA00001-2, Proteintech, 1:10,000) was added and incubated for 1 h at room temperature. The membrane was washed thoroughly with TBST to remove unbound secondary antibody. Protein bands were visualized using a chemiluminescent substrate, and images were acquired and analyzed.

### 2.6 Flow cytometry

Flow cytometry was performed as described in our previous studies (An et al., 2018a). Briefly, mouse incisor pulp tissue was freshly dissected and dissociated into single cell suspension. Cells were then fixed and immune-stained by primary antibodies, followed by the secondary antibodies. FACS analysis was carried on MACSQuant Analyzer 16 flow cytometer (Miltenyi Biotec.) and data were analyzed using Flowjo software. The following antibodies were used for flow cytometry: IL-1β-PerCP 710 (46-7114-82, Invitrogen, 1:100), CXCL2 (Polyclonal Goat IgG, AF-452-SP, R&D systems, 1:40), R-PE–conjugated Donkey Anti-Goat IgG (SA00008-3, Proteintech, 1:100). At least three independent experiments were performed for each analysis.

### 2.6 Masson’s trichrome staining

Tissues were fixed in 10% formalin, routinely dehydrated, and embedded in paraffin. Sections of 4 um thickness were routinely dewaxed and rehydrated. The slides were immersed in mordant staining solution and incubated in a water bath at 60°C for 1 h, followed by rinsing in running water for 10 min. Celestite Blue Solution was applied dropwise onto the section and incubated for 3 min. Slides were lightly washed twice with distilled water. Mayer Hematoxylin Staining Solution was used dropwise for 2∼3 min, followed by washing with distilled water twice. Slides were counterstained by Acid Alcohol Differentiation Solution briefly and rinsed in a running water for 10 min. Ponceau-Acid Fuchsin Solution was applied dropwise for 10 min, followed by washing twice by distilled water. Slides were then incubated with Phosphoplatinic Acid Solution for 10 min, then Aniline Blue Staining solution was applied dropwise for 2 min. Slides were washed with weak acid solution and incubated for another 2 min. Slides were then subsequently dehydrated with 95% ethanol for 30 s, Absolute ethanol twice for 1 min and xylene twice for 1–2 min. Sections were mounted, and cover slipped using resinene (Solarbio, G1346).

### 2.7 Picro sirius red staining

Mouse incisor sections were routinely deparaffinized and rehydrated. Iron Hematoxylin Staining Solution was prepared in advance and applied dropwise on the sections for 5–10 min. Excess staining solution was removed by rinsing the slides with distilled water for 10–20 s followed by washing under tap water for 5–10 min. Sirius Red Staining Solution (Solarbio, G1473) was used dropwise for 20 min, and rinsed slightly under running water to remove the excess staining solution. Slides were then subsequently dehydrated with 95% ethanol for 30 s, Absolute ethanol twice for 1 min and xylene twice for 1–2 min. Sections were mounted, and cover slipped using resinene.

### 2.8 Statistical analysis

All data were obtained from multiple independent biological samples (n> = 3). Statistical analyses were performed using GraphPad Prism 8.0 software. Unpaired t-tests to compare differences between the two groups. Data are presented as the mean ± SEM. Results with *p* < 0.05 were considered statistically significant (*p < 0.05, **p < 0.01, ***p < 0.001).

## 3 Results

### 3.1 Single-cell transcriptome analysis reveals aging-related phenotypes in dental pulp

**FIGURE 1:**
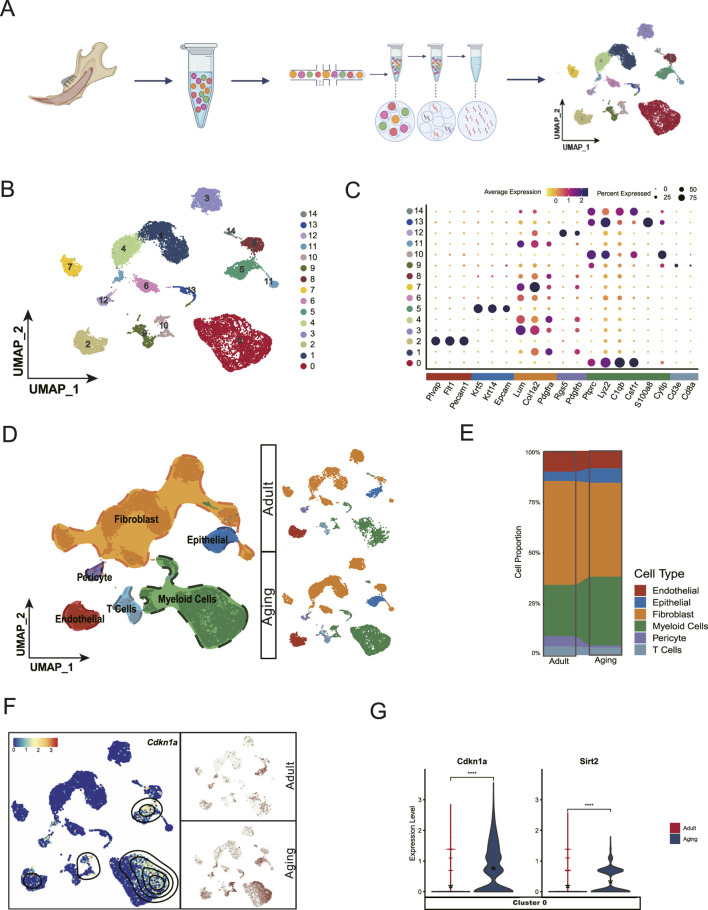
Single-cell landscape of dental pulp aging. **(A)** Schematic workflow of single cell collection and data analysis. **(B)** Uniform Manifold Approximation and Projection (UMAP) visualization classifies the entire dental pulp cell population into 15 distinct clusters. **(C)** Dot plot showing the expression profile of the representative cell-type-enriched marker genes across all 15 clusters. The node size corresponds to the percentage of cells expressing each marker, while color intensity reflects gene expression levels (low to high). **(D)** UMAP visualization identifies six major subpopulations based on distinct cell type markers. **(E)** Cell proportion analysis shows that fibroblasts and myeloid cells are the predominant cell types in the pulp atlas, suggesting their potential roles in pulp tissue maintenance. **(F)** Cdkn1a, a marker of cell senescence, shows elevated expression levels in a specific subpopulation of myeloid cells with aging. **(G)** Violin plot showing the increased expression levels of Cdkn1a and Sirt2 inCluster 0 myeloid cells in the aging group compared to young adult counterparts.

Senescence is a complex process characterized by changes in gene expression and cellular function as individuals age (Zhang et al., 2020). To uncover the molecular mechanisms underlying aging in dental pulp, we collected whole pulp cells from aged mice and performed single-cell RNA sequencing using 10X Genomics technology. Additionally, we also integrated publicly available data from adult incisors of mice aged between 2 and 4 months for parallel analysis (Figure 1A). After rigorous quality control, our dataset comprised 12443 cells from the aged group and 5,696 cells from the adult group for comparative analysis. We employed UMAP for two-dimensional visualization, categorizing mouse dental pulp cells into 15 distinct subpopulations (Figure 1B). Based on specific marker gene expressed in these subpopulations, we identified six main cell types: Fibroblasts (Lum^+^, Colla2^+^) (Guerrero-Juarez et al., 2019), Myeloid cells (*Lyz2* ^*+*^, *C1qb* ^*+*^, *Csf1r* ^*+*^ and Ptprc^+^) (Zhu et al., 2022), T Cells (*Ptprc* ^*+*^, *Cd3e* ^*+*^), Endothelial (*Plvap* ^*+*^, *Flt1* ^*+*^, *Pecam1* ^*+*^), Epithelial (*Krt5* ^*+*^, *Krt14* ^*+*^, *Epcam* ^*+*^), and, Pericytes (*Rgs5* ^*+*^, *Pdgfrb* ^*+*^) (Figures 1C, D). Notably, Fibroblasts and Myeloid cells predominated in the pulp atlas (Figure 1E), suggesting their key roles in maintaining pulp tissue homeostasis.

Aging-related gene markers, particularly from the Cdk inhibitor and Sirt gene families, are well-studied for their roles in cellular senescence and longevity (López-Otín et al., 2023; Ji et al., 2022). Cellular senescence is characterized by a decline in proliferative capacity (Hayflick and Moorhead, 1961), often resulting from cell cycle arrest mediated by the accumulation of CDKN1A (Engeland, 2022). Sirtuins, a family of genes (SIRT1 to SIRT7 in mammals), are critical regulators of metabolism, DNA repair, stress responses, and longevity (Houtkooper et al., 2012).

Our single-cell datasets revealed elevated expression of *Cdkn1a* and *Sirt2* in aged myeloid cells, predominately within Cluster 0 myeloid cells., These findings suggest that myeloid cells may be more susceptible to the early stage of aging (Figures 1D–G).

### 3.2 Aging preferentially impacts Ccrl2+ macrophages

**FIGURE 2:**
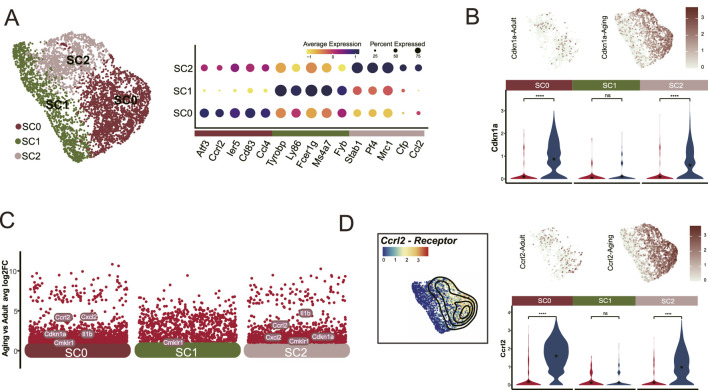
*Ccrl2* ^+^ Macrophages are significantly affected by aging. **(A)** Segmentation of the myeloid cells population (Cluster 0) into three distinct clusters: SC0, SC1 and SC2, showing marked upregulation of Cdkn1a in SC0 and SC2. **(B)** DEG analysis identifies Ccrl2 as one of the top upregulated gene in SC0 and SC2 myeloid cells in aged pulp compared to young adults. **(C)** UMAP and Violin plot analysis illustrated the differential expression of Ccrl2. **(D)** Dot plot showing the expression patterns of cell-type-enriched marker genes across the three subclusters.

To identify which myeloid cell populations are most impacted by aging, we categorized these cells into three distinct subclusters: SC0, SC1, and SC2 based on their prominent DEGs. SC1 was characterized by monocyte markers, including *Ly86*, *Fcer1g*, *Ms4a7*, and *Fyb*. Conversely, SC0 and SC2 were identified as macrophage populations based on the expression of *CD83*, *Atf3*, *Stab1Stab1*, and *Mrc1* (Figure 2A).

Notably, both SC0 and SC2 exhibited a marked increase in *Cdkn1a* expression (Figure 2B). Further scrutiny of DEGs revealed a significant elevation in *Ccrl2* ^*+*^ expression within these subclusters (Figures 2C, D) which correlated with the elevated *Cdkn1a* levels (Figure 2B). This finding highlights the susceptibility of these specific myeloid cell clusters to aging. Based on these observations, we identified SC0 and SC2 cells as *Ccrl2* ^*+*^ Macrophages and demonstrated their vulnerability to aging. These results suggest a potential role for *Ccrl2* ^*+*^ macrophages in the aging process.

### 3.3 Enhanced chemotaxis signals transmitted from fibroblast to macrophages via the CCRL2/RARRES2/CMKLR1 axis

**FIGURE 3:**
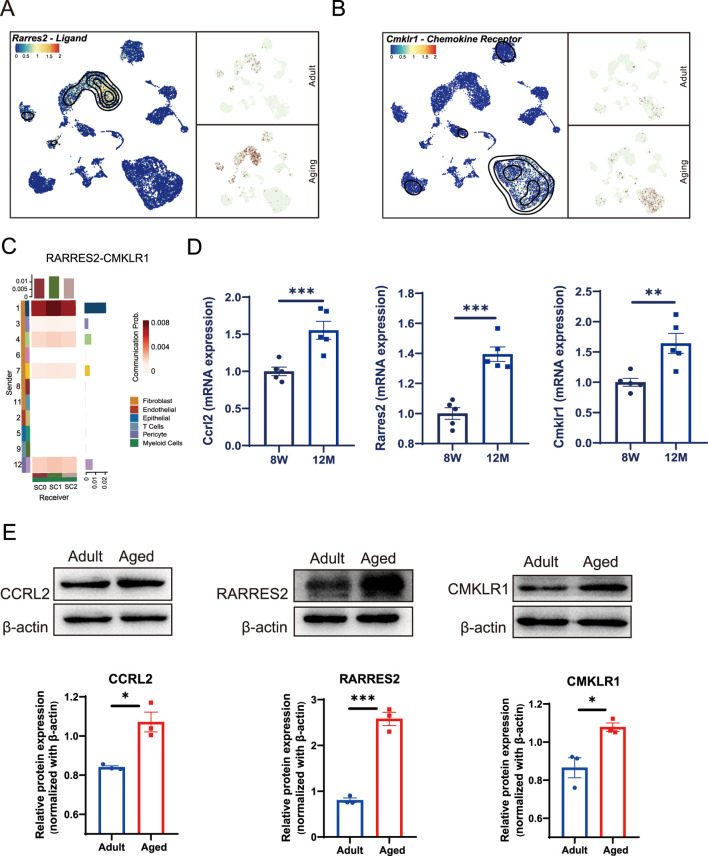
Chemotactic signals transmitted between fibroblasts and macrophages through Rarres2/Ccrl2/Cmklr1 Axis. **(A)** UMAP analysis reveals an age-associated increase in Rarres2 expression in fibroblast populations. **(B)** Cmklr1 expression is consistently observed across all three subsets of myeloid cells during aging. **(C)** Heatmaps depicting the communication probability between fibroblasts and the other pulp cell populations, mediated by RARRES2 and CMKLR1 interaction. **(D)** Bar plots showing the mRNA expression level of Ccrl2, Rarres2 and Cmklr1 in mouse incisor pulp from adult and aging groups by qPCR quantification. Sample size, n = 5. ** and *** indicate *p* < 0.01 and *p* < 0.001 respectively. **(E)** Western blot analysis identifies enhanced expression levels of CCRL2, RARRES2, and CMKLR1 in the aged groups. The corresponding quantification of each protein’s expression is shown in the lower panels. Sample size, n = 3. * and *** indicate *p* < 0.05 and *p* < 0.001 respectively.

Previous studies have shown that CCRL2 can effectively bind to its specific ligand, RARRES2 (Al Delbany et al., 2021; Bufano et al., 2022), facilitating its presentation to CMKLR1, a chemokine receptor expressed on recipient cells (Ben Dhaou et al., 2021; Zabel et al., 2008). This interaction is crucial for the chemotaxis of macrophages and natural killer (NK) cells (Mattern et al., 2014; Wittamer et al., 2003). To investigate this pathway further, we measured the expression of *Rarres2* and *Cmkrl1* in dental pulp cells derived from both young and aged mice. Our results revealed that *Rarres2* expression was predominantly localized to fibroblasts (Figure 3A), whereas *Cmklr1* was primarily expressed in myeloid cells (Figure 3B) within the aged pulp tissue. The ligand-receptor interaction analysis indicated a strong interaction between *Rarres2* and *Cmklr1*, specifically localized in Cluster 1 fibroblasts and macrophages, respectively (Figure 3C). To validate these findings, we performed qPCR and Western Blot analysis on pulp tissue collected from 8-week-old (young) and approximately 12-month-old mice (aged). Both analyses confirmed an age-related increase in the expression levels of these genes and proteins (Figures 3D, E). The findings demonstrate specific cellular and molecular alterations in the dental pulp during aging,and suggest a functional relationship between fibroblasts and macrophages via the CCRL2/RARRES2/CMKLR1 axis. This interaction may contribute to the altered immune landscape observed in aged dental pulp.

### 3.4 Chemotactic protein secreted by fibroblast progenitors recruit macrophages to stem cell niches for activation and differentiation

**FIGURE 4:**
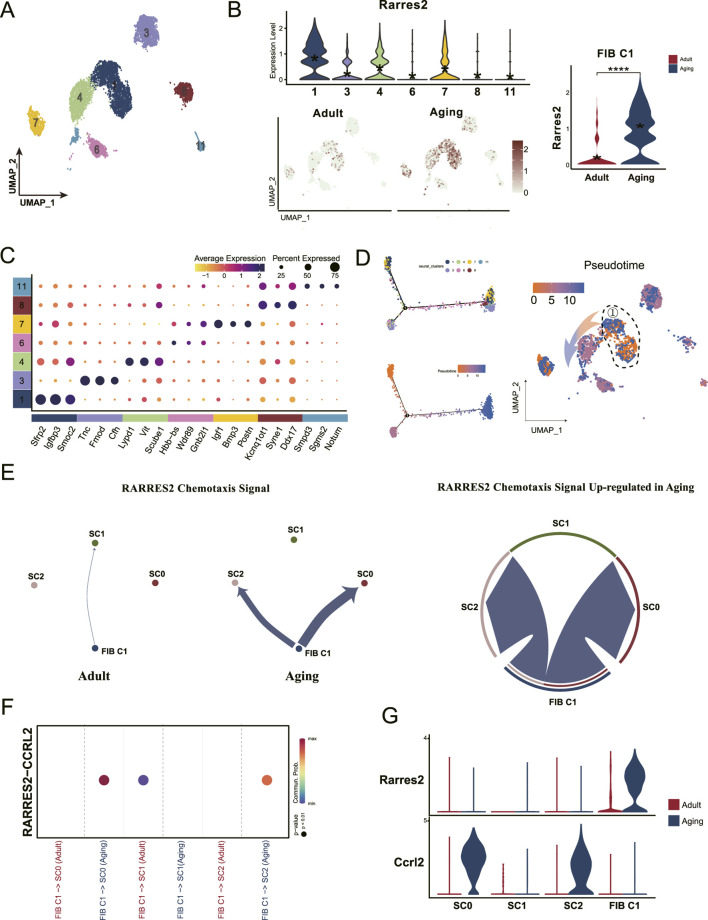
Dental pulp progenitors recruit macrophages through chemotactic protein secretion during aging. **(A)** UMAP analysis classifies all fibroblasts into seven distinct subclusters. **(B)** Violin plots and UMAP visualizations show high Rarres2 expression in the fibroblast, particularly enriched in the Cluster 1 fibroblast (FIB C1) subset. **(C)** Dot plot displaying the expression profiles of cell-type-specific marker genes across the seven fibroblasts subclusters. **(D)** Monocle pseudotime trajectory analysis showcases the developmental progression of fibroblasts, with the pseudotime gradient represented by a color shift from orange (origin) to blue (termination). **(E)** Shell diagram depicting the intensity of RARRES2-mediated chemotactic signaling, highlighting increased signal strength from Cluster 1 fibroblasts towards SC0 and SC2 in the aging group. **(F)** Dot plot emphasizing the enhanced communication likelihood between FIB C1 and Ccrl2+ macrophages (SC0 and SC2), facilitated by the RARRES2-CCRL2 interaction. **(G)** Violin plots showing the expression levels of Rarres2 and Ccrl2 within the myeloid cell subclusters and FIB C1 populations.

To identify which fibroblast group was most influenced by macrophages, we categorized all fibroblasts into seven distinct clusters (Figure 4A), and observed a significant upregulation of *Rarres2* in Cluster 1 (C1) fibroblasts (Figure 4B). Further analysis of DEGs revealed that *Smoc2*, *Sfrp2*, and *Igfbp3* were the most prominent DEGs in C1 (Figure 4C). This finding aligns with previous studies, which identified these genes as markers of a fibroblast subset restricted to progenitor states (Krivanek et al., 2020). Subsequent cell trajectory analysis confirmed that C1 cells represent fibroblast progenitors capable of differentiating into mature fibroblast population (Figure 4D). Ligand-receptor interactions analysis revealed increased communication between C1 fibroblast progenitors and *Ccrl2*-positive macrophages via the RARRES2-CCRL2 signaling pathway (Figures 4E–G). This interaction is critical because RARRES2 complexes are presented to CMKLR1, another chemotactic receptor exclusively expressed across all macrophage clusters (Figure 3B). These findings suggest that the RARRES2 secreted by C1 fibroblast progenitors acts as a chemotactic signal, initiate chemotaxis signal transduction and driving the recruitment of *Ccrl2*-positive effector macrophages to stem cell niches. This recruitment process may subsequently enhance macrophage activation and promote monocyte to macrophage differentiation within the stem cell niches, potentially influencing stem cell functionality and tissue homeostasis.

### 3.5 Elevated pro-inflammatory factors produced by macrophages in stem cell niches contribute to pulp fibrosis

**FIGURE 5:**
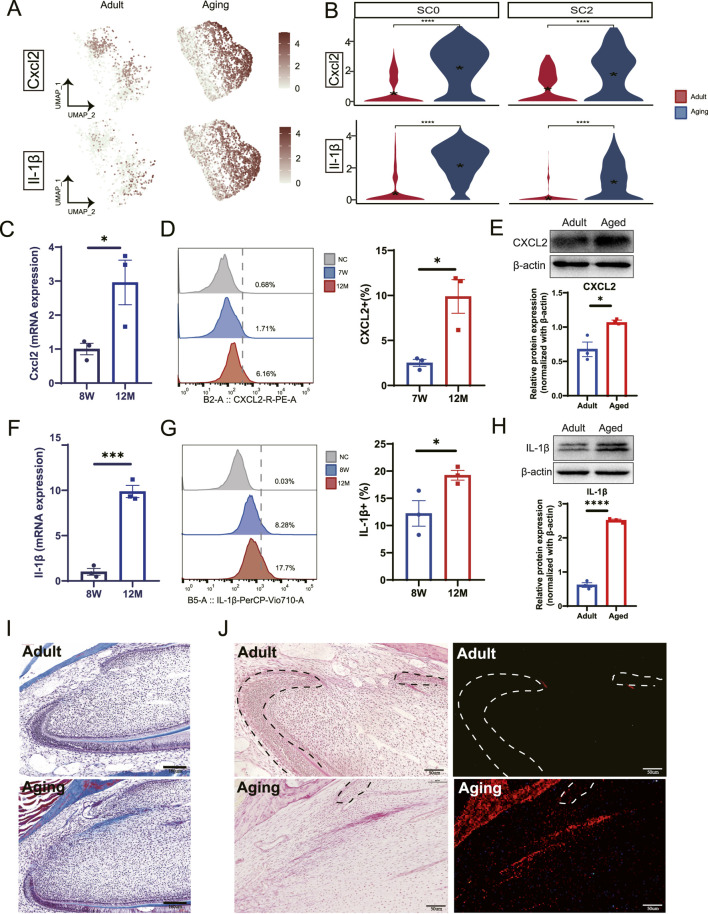
Macrophages secret pro-inflammatory factors contributing to dental pulp fibrosis. **(A)** DEGs analysis combined with UMAP visualization highlight the upregulation of Cxcl2 and Il-1β in aged samples. **(B)** Violin plots reveal the increased expression levels of Cxcl2 and Il-1β in the SC0 and SC2 myeloid subclusters. **(C)** qPCR analysis demonstrates elevated expression of Cxcl2 in mouse incisor pulp tissue from young adults (8 weeks) and aging groups (12 months). **(D)** Flow cytometry analysis quantifies the expression of CXCL2 in mouse incisor pulp across control, 7-week (7 W) and 12-month (12 M) groups (left panel). A corresponding bar plot shows a relative increase in expression in aged pulp tissue compared to young pulp (right panel). **(E)** Western blot demonstrates the increased expression of CXCL2 in aged pulp tissue. The quantification of expression is shown in the lower panel. **(F)** qPCR analysis identifies elevated Il-1β expression in aged mouse incisor pulp tissue. **(G)** Flow cytometry analysis detects IL-1β expression in mouse incisor comparing young with aged pulp tissues. The quantifications of expression levels are shown in the right panel. **(H)** Western blot demonstrates the increased expression of IL-1β in aged pulp tissue. The quantification of expression is shown in the lower panel. **(I)** Masson’s trichrome staining analysis illustrates an increase in collagen fiber bundle in the cervical loop region of aging groups compared to young adult counterparts. Scale bars represent 160 µm. **(J)** Picrosirius Red stains (left panels) and polarized light visualization (right panels) reveal pronounced collagen fibers in the cervical loop region of aging groups compared to young adults. Scale bars are 50 µm. **(C–H)** Sample size, n = 3. *, *** and **** indicate *p* < 0.05, *p* < 0.001 and P< 0.0001 respectively.

To investigate the influence of macrophage recruitment and activation within stem cell niches, we analyzed the expression of key DEGs. Our findings revealed elevated expression of chemokine and cytokine genes, including Cxcl2 and IL-1β, particularly in *Ccrl2* ^+^ macrophages (Figures 5A, B). To validate these results, we extracted total RNA from dental pulp tissues of both young adults (8 weeks) and aged mice (12 months). This analysis demonstrated significant upregulation of *Cxcl2* and *Il-1β* in aged dental pulp (Figures 5C, F). Protein-level validation using flow cytometry (Figures 5D, G) and Western blot analysis (Figures 5E, H) further confirmed elevated levels of these molecules, underscoring the presence of an enhanced pro-inflammatory immune microenvironment within aged pulp that may impair pulp cell function.

Elevated pro-inflammatory factors have been strongly associated with fibrosis in various tissues and diseases contexts (Nie et al., 2023; Trachalaki et al., 2021; Ge et al., 2021). Given the well-established connection between chronic inflammation and tissue fibrosis, we propose that macrophage recruitment and activation by pulp progenitors contribute to pulp fibrosis. To assess the extent and distribution of pulp fibrosis, we performed Masson’s trichrome stain and Picrosirius Red Staining. This analysis revealed increased fibrosis in both the labial and lingual cervical loop regions (Figures 5I, J), previously identified as stem cell niches (An et al., 2018a; Krivanek et al., 2020; An et al., 2018b). Our findings suggest that pro-inflammatory factors derived from macrophages play a critical role in stem cell niche fibrosis within dental pulp during the aging process, potentially disrupting stem cell function and overall tissue homeostasis.

## 4 Discussions

Like other adult stem cells in the body, dental pulp stem cell aging results in diminished regenerative potential, increased susceptibility to dentin damage, and heightened risk of infection. In this study, we employ single-cell transcriptome analysis, complemented by rigorous experimental validation, to decipher the intricate molecular landscape and intercellular communication among various pulp cell populations during the aging process. Our results demonstrate a cascade of events whereby chemotactic proteins secreted by dental pulp progenitors recruit *Ccrl2*-positive macrophages to stem cell niches, subsequently triggering macrophage activation and expansion via the RARRES2/CCRL2/CMKLR1 axis. Further analysis revealed that the secretion of proinflammatory factors by macrophages at the stem cell niches leads to pulp fibrosis and may ultimately cause deterioration of stem cell function during aging.

RARRES2 (Retinoic Acid Receptor Responder 2) encodes a secreted chemotactic protein called Chemerin, which induces chemotaxis via the CMKLR1 G protein-coupled receptor. Notably, we identify elevated expression of Rarres2 in aged dental pulp progenitors for the first time. Chemerin, as the sole ligand for CCRL2 (Bufano et al., 2022), undergoes proteolytic cleavage at its carboxyl terminus, generating various polypeptides with potent chemotactic activity for monocytes/macrophages, myeloid and plasmacytoid dendritic cells, and NK cells (Al Delbany et al., 2021).

CCRL2 binds Chemerin with high affinity without directly inducing downstream signaling (Ben Dhaou et al., 2021; Zabel et al., 2008). Knockdown of CCRL2 significantly reduces Chemerin accumulation (Liang et al., 2019). The interaction between CCRL2 and Chemerin facilitates its presentation to CMKLR1-expressing recipient cells, thereby orchestrating chemotaxis. This mechanism enhances the local concentration of Chemerin, regulating its bioavailability and promoting macrophage and NK cell recruitment (Al Delbany et al., 2021; Ben Dhaou et al., 2021; Zabel et al., 2008). Our data reveal that *Ccrl2* is expressed within a specific subpopulation of macrophages that are attracted to *Rarres2*-expressing fibroblast progenitors. These protein complexes are subsequently presented to *Cmklr1*-expressing macrophages, a feature observed across all macrophage populations. Notably, Chemerin activates CMKLR1, driving macrophage chemotaxis and promoting polarization towards the pro-inflammatory M1 phenotype (Ji et al., 2022) (Mattern et al., 2014; Wittamer et al., 2003). This polarization results in the increased secretion of pro-inflammatory cytokines, including IL-1, IL-18, TNF-α, IL-6, and IL-12 (Wittamer et al., 2003; Liang et al., 2019), which playing pivotal roles in regulating inflammatory responses and immune cell activity.

Among the three known Chemerin receptors: CCRL2, CMKLR1, and GPR1 (Bufano et al., 2022), and GPR1 is primarily expressed in the central nervous system and skin (Bufano et al., 2022; Bondue et al., 2011), with no expression observed in dental pulp in our dataset. Therefore, our focus remains on Chemerin’s interactions with CCRL2 and CMKLR1. In addition to its role in immune cell chemotaxis, the Chemerin/CMKLR1 interaction has been implicated in angiogenesis inhibition. For instance, overexpression of active Chemerin proteins in mice in reduces retinal vascular density and tumor graft angiogenesis (Ben Dhaou et al., 2022). Tissue fibrosis has also been linked to vascular dysfunction, with aging-associated impairments in transcription factor like ETS-related gene (ERG) leading to decreased vascular repair and persistent fibrosis in lung injury models (Caporarello et al., 2022). Given the critical role of blood vessels in dental pulp, their potential association with pulpal fibrosis remains unexplored. Our single-cell analysis revealed a decreased frequency of endothelial cells and pericytes in aged pulp (Figure 1E). Suggesting impaired vasculature. These findings highlight the need for future studies to investigate the interplay between Chemerin/CMKLR1, pulpal vasculature, and fibrosis to better understand their collective impact on dental pulp aging and health.

Although our study utilized dental pulp from mice aged approximately 12 months, true aging in dental pulp may occur later, at 18–26 months (Omrani et al., 2023; Wang et al., 2023). Despite this limitation, our single-cell data provide valuable insights into the early molecular changes associated with dental pulp aging. Further studies employing lineage tracing of dental pulp progenitors will be essential to further validate their stem cell properties and functional roles during aging.

## 5 Conclusion

In summary, our study unveils the intricate crosstalk between dental pulp progenitors and inflammatory macrophages, which potentially initiates and exacerbates pulpal fibrosis during aging. These findings establish a novel theoretical foundation and offer a promising treatment avenue for aging-related pulpal fibrosis, providing potential solutions for dental issues associated with aging.

Furthermore, our parallel studies comparing mouse and human pulp cells at the single-cell level uncover conserved interaction between fibroblasts and immune cells. This finding highlights the relevance and translational applicability of mouse models to human studies. Further investigation into the specific mechanisms by which pro-inflammatory factors drive fibrosis are essential. Such research could unravel the complexities of pulpal aging and pave the way for more effective strategies to combat age-related dental decay and dysfunction in humans.

## Data Availability

Single-cell RNA-Seq data for aged dental pulp is available under the access number GSE253259 in the Gene Expression Omnibus (GEO) database. Additionally, data on dental pulp aged between 2 to 4 months, classified as “Adult”, can be obtained from the dataset GSE146123 in the same database.
